# Access, timing and frequency of very early stroke rehabilitation – insights from the Baden-Wuerttemberg stroke registry

**DOI:** 10.1186/s12883-016-0744-7

**Published:** 2016-11-16

**Authors:** Björn Reuter, Christoph Gumbinger, Tamara Sauer, Horst Wiethölter, Ingo Bruder, Curt Diehm, Peter A. Ringleb, Werner Hacke, Michael G. Hennerici, Rolf Kern, and Stroke Working Group of Baden-Wuerttemberg

**Affiliations:** 1 Department of Neurology and Neurophysiology, University Hospital Freiburg, Breisacher Straße 64, 79106 Freiburg, Germany; 2 Department of Neurology, University Hospital Heidelberg, Heidelberg, Germany; 3 Department of Neurology, Universitätsmedizin Mannheim, University of Heidelberg, Mannheim, Germany; 4 formerly affiliated to Department of Neurology, Bürgerhospital, Stuttgart, Germany; 5 Office for Quality Assurance in Hospitals (GeQiK), Baden-Wuerttembergische Hospital Association, Stuttgart, Germany; 6 Department of Internal/Vascular Medicine, Max-Grundig-Klinik, Bühl, Germany; 7 Department of Neurology, Klinikum Kempten-Oberallgaeu, Kempten, Germany

**Keywords:** Acute stroke, Rehabilitation, Physical therapy, Occupational therapy, Speech therapy, Stroke unit concept

## Abstract

**Background:**

While the precise timing and intensity of very early rehabilitation (VER) after stroke onset is still under discussion, its beneficial effect on functional disability is generally accepted. The recently published randomized controlled AVERT trial indicated that patients with severe stroke might be more susceptible to harmful side effects of VER, which we hypothesized is contrary to current clinical practice. We analyzed the Baden-Wuerttemberg stroke registry to gain insight into the application of VER in acute ischemic stroke (IS) and intracerebral hemorrhage (ICH) in clinical practice.

**Methods:**

99,753 IS patients and 8824 patients with ICH hospitalized from January 2008 to December 2012 were analyzed. Data on the access to physical therapy (PT), occupational therapy (OT), and speech therapy (ST), the time from admission to first contact with a therapist and the average number of therapy sessions during the first 7 days of admission are reported. Multiple logistic regression models adjusted for patient and treatment characteristics were carried out to investigate the influence of VER on clinical outcome.

**Results:**

PT was applied in 90/87% (IS/ICH), OT in 63/57%, and ST in 70/65% of the study population. Therapy was mostly initiated within 24 h (PT 87/82%) or 48 h after admission (OT 91/89% and ST 93/90%). Percentages of patients under therapy and also the average number of therapy sessions were highest in those with a discharge modified Rankin Scale score of 2 to 5 and lowest in patients with complete recovery or death during hospitalization. The outcome analyses were fundamentally hindered due to biases by individual decision making regarding the application and frequency of VER.

**Conclusions:**

While most patients had access to PT we noticed an undersupply of OT and ST. Only little differences were observed between patients with IS and ICH. The staff decisions for treatment seem to reflect attempts to optimize resources. Patients with either excellent or very unfavorable prognosis were less frequently assigned to VER and, if treated, received a lower average number of therapy sessions. On the contrary, severely disabled patients received VER at high frequency, although potentially harmful according to recent indications from the randomized controlled AVERT trial.

## Background

Organized stroke unit care has proven to be more effective than treatment on general wards only [1–3]. A systematic review of the Stroke Unit Trialists Collaboration demonstrated that professional stroke unit care is particularly superior because it prevents immobility-related complications. Further, physical therapy (PT), occupational therapy (OT) and speech therapy (ST) including are regarded to significantly account for this effect [4]. Dysphagia is a common finding in stroke and associated with a higher risk for pneumonia, malnutrition and death, thus making a swallowing assessment necessary immediately after admission by trained nursing staff and/or speech therapists [5, 6]. Recently a Cochrane review confirmed the general effectiveness of physical rehabilitation on stroke outcome and also provided some evidence for beneficial effects when treatment was initiated early, although a lack of high quality studies was noted [7]. Very early rehabilitation (VER) is thought to prevent inactivity-associated complications of multiple biological systems, i.e. the respiratory system (pneumonia, atelectases), blood circulation (deep vein thrombosis, pulmonary embolism), immunosuppression, bedsores and catabolism/muscle atrophy. Further favourable effects are hypothesized to comprise of stimulated neuronal plasticity and a reduced risk for mood disorders associated with stroke [8–11]. The benefits of VER are however accompanied by potentially harmful side effects as arterial blood pressure oscillation during exercise and/or out of bed mobilization with a higher risk for hematoma growth in intracerebral hemorrhage (ICH), collapsing collateral blood supply of the penumbra in ischemic stroke (IS), and secondary ICH in successfully recanalized IS patients [12–14]. However, the evidence for both the precise beneficial and harmful effects of VER remains poor [15]. In April 2015 the phase III randomized controlled “Efficacy and safety of very early mobilization within 24 h of stroke onset (AVERT)” trial was published and demonstrated a lesser chance to achieve functional independence 3 months after stroke in the earlier and more intensively treated stroke group, defined as a modified Rankin scale (mRS) of 0–2. Subgroup analyses indicated that patients with severe stroke and/or ICH might be more susceptible to harmful side effects [16]. Multiple factors might have contributed to these findings. The treatment group was mobilized earlier and received twice as much daily sessions of out of bed therapy, which took three times longer than for the control group. Despite to this uncertainty regarding the harmful factor, it is somewhat unclear to what extend regular stroke care will have to change in light of the AVERT findings. This moreover since there is a lack of description of interdisciplinary therapy content (type, frequency and intensity) and therapy strategy (in-bed therapy vs. out-of bed mobilization) in everyday clinical practice [16]. Based on the authors clinical impressions we hypothesized a positive correlation between stroke symptom severity and VER at higher frequency, which might need reevaluation after publication of the AVERT trial. With a descriptive analysis of the Baden-Wuerttemberg (BW) stroke registry we thus aimed to provide a more detailed insight into the application of PT, OT, and ST in patients with IS and ICH in clinical practice.

## Methods

We performed a retrospective analysis of patients with acute IS and ICH prospectively registered in a large and consecutive hospital-based stroke registry in central Europe. Permission to analyze the registry was obtained by the governing board of the office for quality assurance in hospitals (Geschäftsstelle für Qualitätssicherung im Krankenhaus, GeQik).

### Setting, eligibility criteria and study size

BW is Germany’s third largest federal state regarding size and population with 35,742 km^2^ and 10.8 million inhabitants. In 1998 BW implemented a structured three-level medical concept for the treatment of stroke [17]. Today about 140 hospitals are involved in acute stroke care, comprising of stroke centers, hospitals with regional or local stroke units, and hospitals without a specific stroke unit. The quality of acute stroke treatment is monitored since 2004. Transfer of pseudonymized data on baseline and treatment characteristics to the Office for Quality Assurance in Hospitals is stipulated by federal state law.

**Fig. 1:**
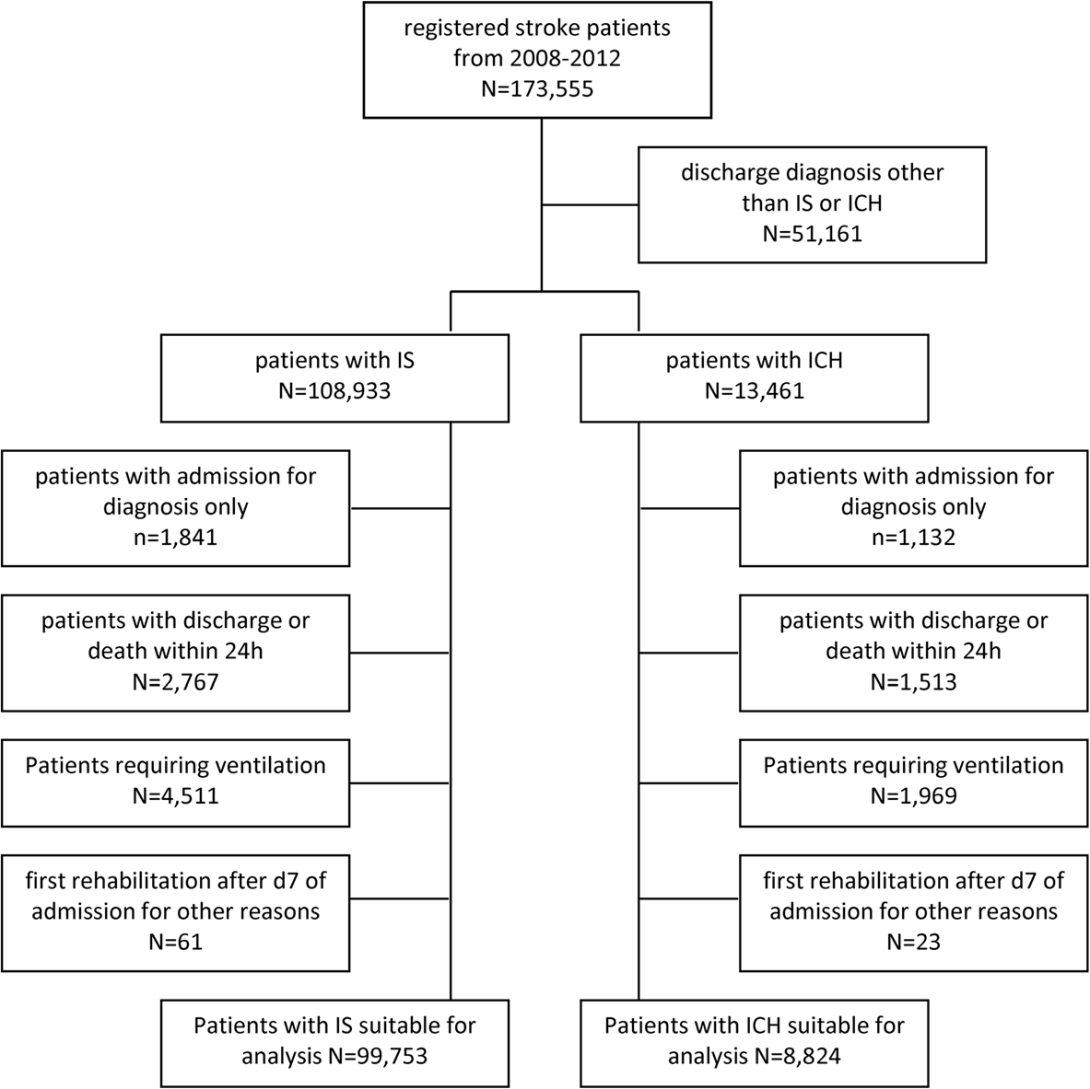
Study cohort selection flow diagram

Data covering a period of 5 years, from January 1, 2008 to December 31, 2012 were analyzed in the present study. 99,753 patients with IS and 8824 patients with ICH were selected out of 122,394 patients with discharge ICD10 diagnosis I61 (nontraumatic ICH) or I63 (IS). 9180 patients with IS and 4637 patients with ICH were excluded from our analysis according to the following criteria, making sufficient participation in VER unlikely (Fig. 1): diagnosis and treatment in emergency departments only (*N* = 1841 [IS] and *N* = 1132 [ICH]); discharge from in-hospital care or death within 24 h (*N* = 2767 [IS] and *N* = 1513 [ICH]); patients requiring mechanical ventilation, reported as “during hospitalization” from 2008 to 2009 and reported as “within 24 h after admission” from 2010 to 2012 (*N* = 4511 [IS] and *N* = 1969 [ICH]); and first rehabilitation therapy > 7 days of admission for other reasons (*N* = 61 [IS] and *N* = 23 [ICH]). Baseline characteristics and data on PT, OT, and ST of the remaining patients are described in detail.

### Variables and statistical analysis

Documentation includes patient demographic data, medical history on cardiovascular risk factors (arterial hypertension and hypercholesterolemia documented since 2010) and previous cerebrovascular events, hospital admission time, level of hospital care, admitting department and ward, nature and timing of diagnostic procedures, intravenous thrombolysis, in-hospital complications, discharge information, and hospital mortality. Stroke severity at admission was assessed using the National Institute of Health Stroke Scale (NIHSS) score and modified Rankin Scale (mRS) score. A pre-stroke mRS score was documented at admission to estimate acute deterioration of functional ability. At discharge a final mRS score was determined. Data on paresis, aphasia, and dysarthria was collected based on clinical examination at admission and discharge.

First contact to a therapist was documented in the data collection form in three time intervals for PT (no treatment, <24 h, 24–48 h, >48 h after admission) and two time intervals for OT and ST (no treatment, <48 h, ≥48 h after admission). The number of therapy sessions (PT, OT, and ST) was collected for the first 7 days of hospitalization. Zero therapy sessions were documented for patients being attended once by a therapist but without need for treatment. In case of discharge home, transfer to other facilities or death within 7 days of admission a calculated score had to be extrapolated by the admitting hospital. To provide an example, four therapy sessions within 6 days of hospitalization are calculated as (4:6)*7 = 4.7, rounded five sessions). The registry collects no data on the amount of therapy in minutes or the specific type of therapy, i.e. in-bed or out of-bed therapy.

We used standard descriptive statistics to explore patient characteristics and access to VER stratified by discharge diagnosis IS and ICH and functional disability at admission and discharge. Functional disability was measured as paresis, aphasia, and dysarthria at admission and discharge, and mRS score at discharge. Multiple logistic regression models were then used to assess the association between VER and clinical outcomes. The selected outcome parameters were (A) the mRS score at discharge (ordinal logistic regression analysis), (B) the full recovery from specific disabilities at discharge (paresis, dysarthria, aphasia; binary logistic regression analysis), (C) the risk to develop pneumonia during the hospitalization period (binary logistic regression analysis), and finally (D) the mortality rate (binary logistic regression analysis). The analyses were performed for the stroke etiologies IS and ICH independently and the models were fitted stratified either for the time interval from admission to first therapy session or for the number of therapy sessions during the first 7 days of hospitalization. All analyses were adjusted for patient characteristics (age, sex, pre-stroke and admission mRS scores, NIHSS score at admission, prior stroke event, diabetes, atrial fibrillation), admitting facility, IV thrombolysis, and length of hospital-stay. All statistical tests were two-sided, and *p* values of <0.05 were considered to be statistically significant. The analyses were carried out using SAS 9.3 (SAS Institute Inc., Cary, NC, USA).

## Results

### Patient baseline characteristics

**Fig. 2:**
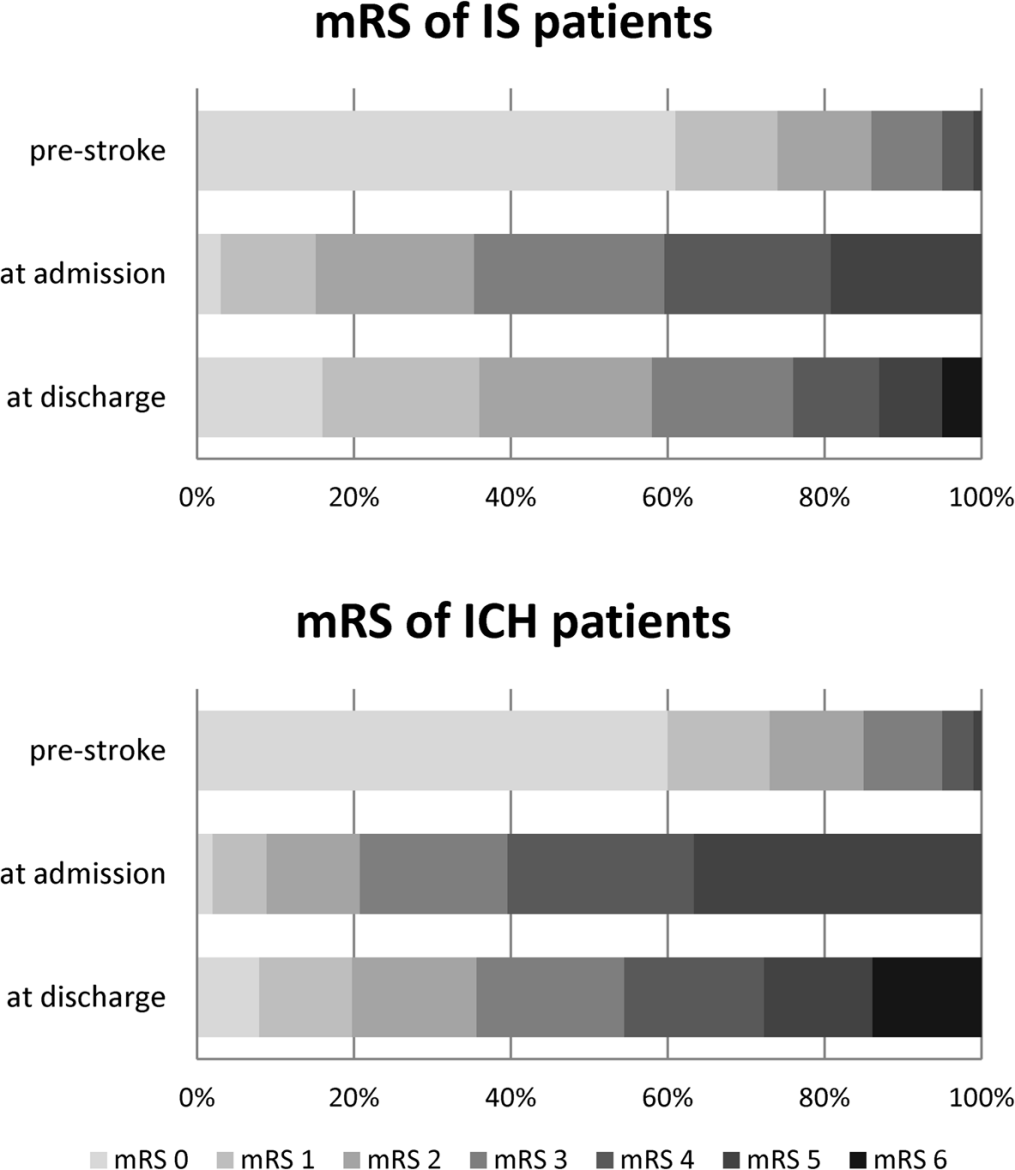
Scores on mRS prior to stroke, at admission, and at discharge for patients with IS and ICH. The mRS score at discharge was missing for *N* = 4221 (4%) of the patients with IS and *N* = 629 (7%) of the patients with ICH

Patient characteristics of the study cohorts are shown in Table 1. The sex ratio and age were equally balanced in stroke patients suffering IS or ICH with approximately 50% being women and a median age of 76 years (interquartile range (IQR) 68 to 83 years). The median NIHSS score in IS was 4 (IQR 2 to 9) and 7 (IQR 3 to 14) in ICH. About two thirds of patients with both IS and ICH presented with a paresis at admission. Aphasia was reported in 30% (IS) and 35% (ICH) and dysarthria in 40% (IS) and 42% (ICH). In both groups 86% were functionally independent previous to acute stroke with a mRS score of 0 to 2 and this number declined to 58% in IS and 36% in ICH at discharge (Fig. 2). Patients with ICH had a higher likelihood to be treated in stroke centers providing maximum care. Nevertheless, approximately 20% of the patients with IS or ICH were treated in hospitals without a stroke unit. Percentages of patients being admitted to specialized stroke units were 71% in patients with IS and 61% in patients with ICH, while 8% of the patients with IS were treated on intensive care units compared to 23% with ICH. Pneumonias occurred in 5% of the patients with IS and 10% with ICH. The median length of hospital stay for IS was 9 days compared to 11 days for ICH.

**Table 1:** Patient characteristics and general stroke care

Variable	IS	ICH
Patients, n	99.753	8.824
Female sex, %	50	49
Age, median (IQR)	76 (68, 83)	76 (68, 83)
NIHSS, median (IQR)	4 (2, 9)	7 (3, 14)
Missing data on NIHSS at admission, %	16	18
Paresis at admission, %	66	67
Missing data on paresis at admission, %	1	3
Aphasia at admission, %	30	35
Missing data on aphasia at admission, %	3	9
Dysarthria at admission, %	40	42
Missing data on dysarthria at admission, %	5	10
Level of stroke care, %		
Center	23	30
Regional	19	18
Local	38	33
Other	21	19
Admitting ward, %		
Stroke unit	71	61
Intensive care unit	8	23
General ward	22	16
Pneumonia, %	5	10
Median length of stay in days (IQR)	9 (6, 13)	11 (7, 16)

Information on arterial hypertenstion and hypercholesterolemia was not routinely documented over the entire study period and is therefore missing for *N* = 37,846 patients. *Abbreviations*: *IQR* interquartile range, *NIHSS* National Institutes of Health stroke scale

### Timing, numbers of therapy sessions and functional disability at discharge

**Fig. 3:**
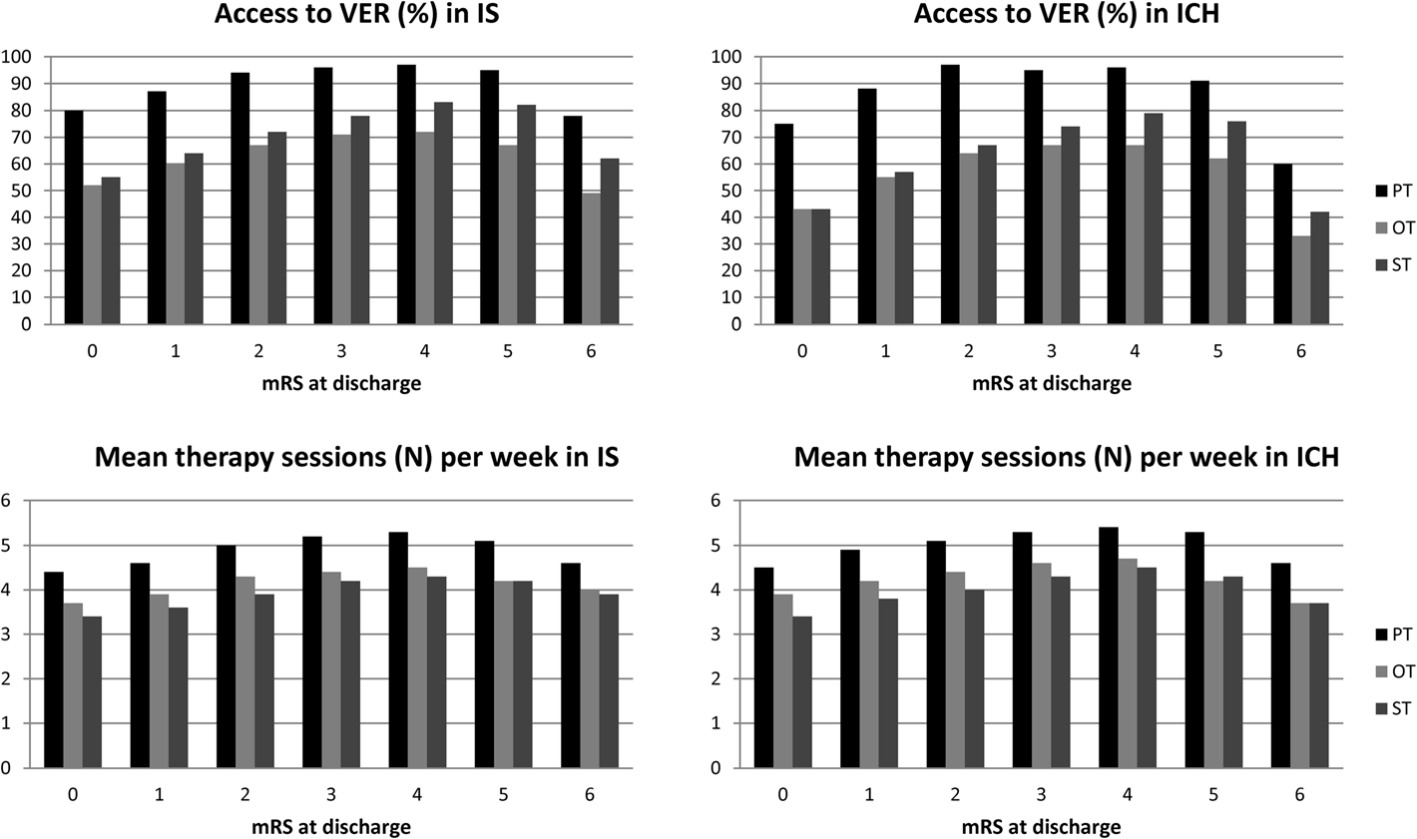
Abbrevations: PT, physical therapy; OT, occupational therapy; ST, speech therapy

90% of the IS patients were attended at least once by a physiotherapist, 63% by an occupational therapist and 70% by a speech therapist (Table 2). When looking exclusively on the treated IS patients 87% received their first PT session within 24 h after admission, 91% their first OT session and 93% their first ST session within 48 h after admission, respectively. The median of therapy sessions during the first 7 days after admission was five for all therapies. The mean numbers provide a more detailed insight, with PT being applied most frequently with 4.9 sessions (standard deviation (SD) 1.8), followed by OT with 4.2 (SD 1.8) units and ST with 4.0 (SD 1.9) sessions. Further stratification according to the mRS score at discharge demonstrated that percentages of treated patients were highest for those with a mRS score of 2 to 5 both regarding the general access to treatment and the average number of treatment sessions (Fig. 3a and c).

**Table 2:** General access to VER, time from admission to first therapy session and average number of therapy sessions

	IS (*N* = 99.753)			ICH (*N* = 8.824)		
Variable	physical therapy, %	occupational therapy, %	speech therapy, %	physical therapy, %	occupational therapy, %	speech therapy, %
No	10	37	30	13	43	35
Yes	90	63	70	87	57	65
< =24 h	87	-	-	82	-	-
24–48 h	9	-	-	13	-	-
< =48 h	-	91	93	-	89	90
> 48 h	4	9	7	5	11	10
Units						
median (IQR)	5 (4, 6)	5 (3, 5)	5 (2, 5)	5 (5, 6)	5 (3, 5)	5 (3, 5)
mean (SD)	4.9 (1.8)	4.2 (1.8)	4.0 (1.9)	5.2 (1.8)	4.4 (1.8)	4.2 (1.8)

Information on units are given only for those patients being attended by a therapist. Numbers of units are reported for the first 7 days of hospital stay. In case the patient was hospitalized for less than 7 days an extrapolated and rounded 7 day-frequency was reported

The proportion of ICH patients being attended by a therapist were 3% less for PT, 6% less for OT, and 5% less for ST, respectively (Table 2). In addition the proportion of patients receiving treatment within the first 24 h (PT) or 48 h after admission (OT, ST) was similarly lower. However, compared to IS the mean number of applied therapy sessions was higher for all therapies (PT 5.2 (SD 1.8) units, OT 4.4 (SD 1.8) units, and ST 4.2 (SD 1.8) units). Stratification according to the mRS score at discharge showed a similar distribution pattern as in IS patients, but revealed lower percentages in particular in those ICH patients with either a very favourable or a very unfavourable outcome (mRS 0 or 6, Fig. 3b and d).

### Access to therapy in specific disabilities related to stroke

The analysis of VER with respect to specific stroke symptoms at admission revealed that ≥95% of the patients with IS or ICH and paresis were treated with PT, while OT was applied less frequently with approximately 70% (Table 3). Compared to IS a lower percentage was observed for ST in patients with aphasia or dysarthria suffering from ICH (87–88% vs. 83–84%).

**Table 3:** Access to therapy for patients with specific disabilities at admission

	IS, %	ICH, %
Paresis at admission		
Physical therapy	96	95
Occupational therapy	71	69
Aphasia at admission		
Speech therapy	88	83
Dysarthria at admission		
Speech therapy	87	84

Data on therapy was missing in IS (group total *N* = 99.753) for *N* = 7.496 (paresis), *N* = 4.186 (aphasia), *N* = 4.619 (dysarthria) and in ICH (group total *N* = 8.824) for *N* = 1.308 (paresis), *N* = 709 (aphasia) and *N* = 771 (dysarthria)

### Effectiveness and mortality

The outcome analyses we hindered by heavily interfering individual decision making of the hospital staff, i.e. patients with rapid improvement of stroke symptoms received therapy less frequently, many patients with foreseeable very poor outcome were either not attended by a therapist or received less therapy sessions, and patients at high risk to develop pneumonia were attended by a speech therapist more frequently. As a consequence and although we`ve adjusted the statistical model for patient characteristics, hospital and treatment parameters as successfully done previously we were finally unable to produce meaningful results (data not shown) [18–20].

## Discussion

Despite to the fact that presumably most hospitalized stroke patients receive VER in a widely varying amount, surprisingly little is known about its current stage in clinical routine [16].

We report data from the Baden-Wuerttemberg stroke registry on the general access to VER, the timing from admission to first physical, occupational, and speech therapy and the average numbers of therapy sessions. All admitted patients with the ICD10 diagnoses I61 and I63 from a 5 year period were taken into analysis, except for patients with either early discharge within 24 h of admission or mechanical ventilation, thereby making the application of VER unlikely. In our study population approximately 80% of the patients admitted for IS or ICH were treated on stroke units or intensive care units with a correspondingly high level of expertise. About 90% of the study cohorts had access to PT and this number was ≥95% for patients with motor deficits at admission. OT was applied in approximately 60% of the total study cohorts. 26% (IS) and 28% (ICH) of the patients with motor deficits at admission were not attended by an occupational therapist. 70% (IS) and 65% (ICH) of the total study population were evaluated and/or treated by speech therapists. For patients with speech or language disorders at admission access to ST was higher, suggesting a moderate undersupply of 10% (IS) to 14% (ICH). The majority of patients with IS and, to a slightly minor degree, ICH received their first PT session within 24 h and OT or ST session within 48 h of hospitalization.

Based on our data VER might be less frequently applied to patients with ICH compared to IS and also the proportion of patients with ICH and immediate access to therapy was observed to be lower. Differences were small but consistent throughout the analysis. Two possible factors might have contributed to this finding. First, patients with fatal ICH were more frequently denied therapy compared to IS patients with the same outcome. This might be due to earlier decision making for palliative care in ICH than in IS. Second, a survey from 2011 indicated that stroke professionals seem to be more restrained regarding early mobilization in patients with ICH for concerns regarding blood pressure oscillation, which also may account for the observed differences [21]. This opinion is supported by observations from a post-hoc analysis of the INTERACT 2 trial that excessive blood pressure variability as it may occur during out-of bed mobilization seems to be associated with poorer outcome [22]. In our analysis a discharge mRS score of 2 to 5 was associated with the highest application of VER both regarding its general access and the average number of sessions for all three types of therapy. This observation was first reported for stroke patients in the 1970s, albeit the availability of PT, OT and ST was much less and the timing from onset to therapy was reported in weeks rather than days [23]. It might also explain the higher average number of therapy sessions in patients with ICH, with patients being more severely disabled compared to IS. We performed multiple logistic regression analyses to investigate the influence of VER on stroke outcome but the results were fundamentally contradictory, most likely because we were finally unable to statistically compensate biases by individual decision making. We thus believe that stroke registries are not suitable to investigate the influence of VER on stroke outcome.

Although the incidence of ischemic and hemorrhagic strokes is high with millions affected worldwide annually, the optimal dose and timing for very early stroke rehabilitation are entirely unclear [24]. Data from animal stroke models revealed a potentially NMDA-mediated increased cell death rate within the infarct and peri-infarct area under very early and intense training, which was counterbalanced by an improved motor performance when therapy started at the earliest 5 days post-stroke [25–29]. However, the intensity of training in animal models is much higher than compared to humans [28]. A trial comparable to animal studies used constraint-induced movement therapy and observed a worse functional day 90 outcome when therapy started at day 10 after stroke onset [30]. VER within 24 h of stroke onset was investigated in three randomized controlled trials of limited size which were published between 2008 and 2010. A systematical review in 2015 did not demonstrate superiority of VER compared to standard care [31–34]. In April 2015 the large phase III AVERT trial was published and to the surprise of the community reported a less favourable 3 month stroke outcome of the earlier and higher-dose treated group. Although the multiple differences between the study groups (earlier mobilization at higher frequency and for a longer period of time) impair the identification of the actually harmful factor, the AVERT trial broke with the widely-accepted assumption that very early and intense mobilization and rehabilitation represents the best medical therapy to promote recovery in all patients with acute cerebrovascular disease [16]. Further stroke subgroup analyses hypothesized that in particular patients with ICH and/or severe disability might benefit from delayed mobilization and rehabilitative treatment at lower dosage. However, even although more than 2000 patients participated in the trial, pre-specified subgroup analyses were still underpowered to draw robust conclusions. Very recent data from AVERT indicates that short and frequent out-of bed mobilization is superior to an increased amount of time spent with out-of bed activity [35]. In this instable phase VER for patients with severe disability might also be applied in a neurosensorial approach, verticalisation by robotic devices, repetitive treatment by motocycle in bed, swallowing and respiratory treatment [36]. While most clinical trials and observational studies focused on mobilization and physical rehabilitation approaches, the impact of ST was less frequently investigated. ST does not require mobilization and thus is not associated with several potentially harmful side effects of out-of bed PT. Two small clinical trials with in total 59 patients suggested that early ST for mild to moderate aphasia within 3 days of stroke onset results in improved outcome after 6 months [37, 38]. Moreover, very early swallowing assessment by trained nurses or speech therapists is of high value to identify patients at risk for aspiration pneumonia and malnutrition [39].

Obviously, interdisciplinary treatment in the acute phase has to be coordinated and adapted to the acute diagnostic investigations and treatments, otherwise VER might not be applied in the adequate moment [36]. It thus seems very likely that specific subgroups concerning stroke etiology, severity of stroke symptoms, comorbidities, and acute stroke therapy like hematoma evacuation, IV thrombolysis, or thrombectomy need to be taken into account for an individualized rehabilitation protocol [40]. An overview on worldwide national stroke guidelines was presented 2015 by Bernhardt et al and demonstrated a widely recommended early mobilization regime (22 out of 30 national guidelines, of these eight recommended mobilization within 24 h after stroke onset). Early rehabilitation is endorsed in 11 national guidelines, i.e. Canada, Croatia, Finland, Germany, Japan, New Zealand, Norway, Scotland, Singapore, South Africa, and the USA [15]. Current US-American guidelines state that VER in the acute care hospital is optional, but all patients should at least undergo rehabilitative assessment prior to discharge to an inpatient rehabilitation care unit [41, 42]. The 2015 updated Canadian stroke rehabilitation guideline was the first to have incorporated findings from the AVERT trial and does no longer recommend frequent out-of bed activity within 24 h after stroke onset [43]. As a supranational committee, the European Stroke Organization guidelines for the management of ischemic stroke from 2008 recommend an early initiation of rehabilitation therapy but do not provide precise time frames [44]. In Germany, treatment on specialized stroke units or intensive care units is remunerated with the procedure code 8–981.x. This includes daily PT, OT, and ST for patients with treatable disabilities and therapy has to be commenced not later than the next day after admission.

Based on the general access to VER and the average numbers of therapy sessions our data from routine hospital care demonstrates that severely disabled stroke patients receive training at high frequency, most likely under the assumption that they will benefit most. We regard our data robust even though the “admission to treatment time” and not “onset to treatment time” was measured, since there is a correlation between stroke severity and lesser onset to hospital admission time [18, 45]. However, the minimal demand of rehabilitation therapy is at least not to be harmful to the treated subject. The results of the AVERT trial should therefore be taken into account for the application of VER in clinical routine to avoid overly intense therapy in severely disabled patients, as done in the current Canadian stroke rehabilitation practice guidelines [43]. One further important lesson from AVERT is the urgent need for further clinical trials which will have to focus on stroke subgroups instead of the entire spectrum of IS and nontraumatic ICH. Otherwise conclusions as from AVERT lead to misunderstanding. The condition to measure the outcome of the rehabilitation treatment needs to include shaping, that means (as shown in the Baden-Wuerttemberg stroke registry) the treatment has to be adapted to the degree of disability of the patient and his rehabilitation potential. It moreover might be best evaluated by individual goal assessment based for instance on the International Classification of Functioning, Disability and Health [46]. In the meantime a temporary international consensus for severely disabled stroke patients and patients with ICH is regarded necessary.

Specific limitations of our study need to be discussed. Firstly, since the data on therapy was collected from a stroke registry, no quantitative (amount of PT, OT, and ST sessions in minutes) or qualitative (in-bed or out of-bed therapy) information was available. This limits the ability to directly compare our results to the AVERT trial. Moreover, in the BW stroke registry the documentation on timing of OT and ST (≤48 h, >48 h) was less precise compared to PT (≤24 h,>24–48 h, >48 h), which limits a detailed comparison for the first 48 h after admission. Secondly, in large stroke registries the general accuracy of results strongly depends on the accuracy of data input at the participating hospitals. In the Baden-Wuerttemberg stroke registry data documentation is usually performed by a physician, diagnosis related groups (DRG) coordinator or trained study nurse, which should ensure a sufficient data quality. The provided data is validated centrally by predefined logic and range checks and annual performance reports including feedback on data quality are provided for each participating hospital. Nevertheless, for patients being discharged within less than 7 days of admission a calculated score had to be extrapolated by the admitting hospital and it is regarded very plausible that the complexity of data input will negatively affect its accuracy. Based on the lower interquartile range of hospitalization time (7 days in ICH and 6 days in IS) approximately 25% of the ICH and IS patients were discharged after less than 7 days of admission. Thus for this fraction the registry has no access to the raw data and data quality cannot be comparably assured. Thirdly, missing data on functional disability at admission might bias the results and thus interpretation of data.

## Conclusion

Patients admitted for IS or ICH in Baden-Wuerttemberg have excellent access to PT, while an approximately 12–17% undersupply of ST and more in particular a 30% undersupply of OT in patients with treatable disabilities was observed. When stratified according to the mRS at discharge, we observed that in acute stroke care the staff decision for VER and its frequency seem to reflect attempts to optimize resources, i.e. those with either very good or very unfavorable prognosis received therapy less frequently. Severely disabled stroke patients received VER at the earliest and treatment sessions were applied at highest frequency. Since there are current concerns how to perform rehabilitation therapy in severely disabled ischemic stroke patients and patients with ICH in the very early, vulnerable phase, a temporary consensus for this subgroup is regarded necessary. Future clinical trials are needed to investigate the response of specific stroke subgroups to VER and the methodology of these trials will have to be fundamentally reconsidered

## Abbrevations

ICH: Intracerebral hemorrhage
IS: Ischemic stroke
OT: Occupational therapy
PT: Physical therapy
ST: Speech therapy
VER: Very early rehabilitation
